# To P or not to P, is that the question? Rethinking experimental design and data analysis to improve biological significance beyond the statistical significance

**DOI:** 10.1590/1678-7757-2019-ed001

**Published:** 2019-10-07

**Affiliations:** 1 Universidade de São Paulo Faculdade de Odontologia de Bauru BauruSão Paulo Brasil Universidade de São Paulo, Faculdade de Odontologia de Bauru, Bauru, São Paulo, Brasil.

In a assay published in Nature, Valentin Amrhein (University of Basel) and its colleagues Sander Greenland (University of California) & Blake McShane (Northwestern University), present a series of arguments against the stablished “P value-based statistical significance dichotomania”.^1^ The authors use some insightful practical examples, such as data related to unintended effects of anti-inflammatory drugs risks, and consider the influence of human cognitive trend to simplistically bucket results into “statistically significant” and “statistically non-significant” categories and to consider it definitely different.

Importantly, the essay clearly states that the authors are not advocating a ban on P values or statistical measures, but that P values should not be treated categorically or to support dichotomization as statistically significant or not. Similarly, a 2016′ statement of the American Statistical Association^2^ warns against the misuse of statistical significance and P values, which include as a recommendation “don't say statistically significant”. On the other hand, John P. A. Ioannidis (Stanford University), argue that “retiring statistical significance would give bias a free pass' and that ‘irrefutable nonsense would rule’”.^3^ The author states that dichotomous conclusions can be useful for pinning down discoveries in different fields, but the analysis of effect sizes “can often be better than determining whether an effect exists”.

While such discussion is at least provoking and mind challenging, this Editorial goal is to take advantage of the “statistical dogma” questioning to draw some attention to steps that precede the statistical analysis and the generation of a given P value. Indeed, a proper study design can even improve the statistical findings power in a measurable way, but also several aspects of study design and the subsequent data analysis may have a significant (forget P values for a moment), but not statistically quantifiable, impact in the data “significance”. The big mistake would be the overvaluation of statistical analysis methods with the undervaluation of experimental design. In the sequence, some practical examples (derived from our research group data) will be used to illustrate how study design can increase the both “statistical significance” and “biological significance”.

Genetic studies are usually based in a classic control approach, were controls and subjects presenting a given condition are compared in regards of the occurrence and frequency of genetic variants. In this context, the P value derived from the unaffected and affected individuals' comparison is essential to draw any conclusion. In such studies, the number of individual in such groups, but also the frequency of the target genetic variation, the frequency of the studied condition, impact the study power and the determination of the P value, and evidently, the conclusions derived from such data. However, experimental design features, apparently incomputable in the study power determination, can also present a significant impact in the analysis outcome. Thus, in this situation, stratified sampling considering these possible confounding factors could balance the study groups and minimize the effect of external variables on the final data analysis.

In the periodontal genetic studies, generally affected individuals (presenting some form of periodontitis) are compared with periodontally healthy subjects.^4^–^7^ However, in this context, the possibility to control microbial exposure by oral hygiene methods interfere with the exposure factor, and consequently a periodontally healthy population is comprised by subjects that properly perform oral hygiene methods as a routine. Therefore, irrespective of the putative susceptible and resistant genotypic nature, such subjects will not develop the disease phenotype due the proper oral care. Indeed, such unique feature clearly differ from the usual characteristics of infectious diseases genetic studies, where affected and unaffected individuals are typically recruited from endemic areas where groups are naturally exposed to a pathogenic challenge, and the resistant and susceptible phenotypes are consequently exposed.^8^,^9^ Therefore, the absence of the microbial factor in a periodontally healthy population, clearly disregard the case-control study architype, which determine if an exposure is associated with an outcome.^8^,^9^

In other words, the absence of an archetypical control with a defined resistance phenotype may limit the odds of the identification of genotypic differences when compared with a susceptible group. In order to adapt the study design to the exposure concept, in periodontitis genetic studies the control group should comprise a microbially exposed group with a distinct phenotypic outcome than chronic periodontitis.^10^ Such features can be found in individuals presenting chronic gingivitis, characteristically exposed to a periodontal microbial challenge associated with a reversible low severity disease form characterized by the absence of attachment, which in theory, represent “resistance” phenotype/genotype. Indeed, the “resistant versus susceptible” phenotype analysis, when compared to the traditional “healthy versus diseased” approach, significantly impacted the study power and odds of identification of genetic factors involved in PD.^10^ The overall impact in the study power was the boost to >85% of a previous <30% power, while the overall odds ratio values seems to double in this approach; being such impact derived from the proper observation of the archetypical study design, supported by exposure-based phenotypes determination, comprehensively used in infectious diseases genetic studies.

Phenotypic variation can also be a supporting factor for data analysis and interpretation in order to determine the possible involvement of a given factor in a pathological process. Still in periodontitis context, molecules that control osteoclastogenesis process have been regarded as potential determinants of disease onset, progression and severity. In this framework, the osteoclastogenic factor RANKL and its endogenous inhibitor OPG, have been in the focus of numerous studies in the field over the last years.^11^ Briefly, RANKL levels are supposed to be locally upregulated by chronic inflammatory immune response elements, leading to the bone resorption that characterizes periodontitis, while anti-inflammatory mediators present a counteracting effect via OPG upregulation. A common approach in host factors focused studies is to comparatively measure the coding mRNA or protein levels, or to perform the staining of cells positive for such targets, in diseased and healthy tissues. However, such approach can be also limiting in terms of data interpretation and “biological significance” by the reasons similar to those highlighted for the genetic studies.

**Figure 1:**
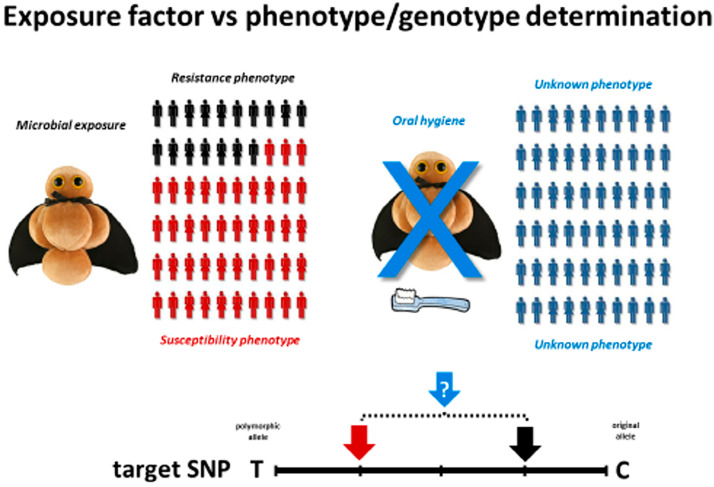
Exposure factor vs phenotype/genotype determination

A healthy tissue represents homeostasis and a diseased tissue represents pathology. However, both allegedly destructive (RANKL) and protective (OPG) factors are upregulated in diseased tissues, similarly to numerous inflammatory and also anti-inflammatory mediators.^11^,^12^ Indeed, one could argue how increased levels of anti-inflammatory and anti-osteoclastogenic factors, associated to “highly significative” P values (in healthy vs diseased tissues comparison), are presented in inflamed and diseased periodontal sites? It is also important to consider that if a given molecule, with unknown role, would identified as upregulated in diseased periodontal tissue, the immediate assumption regarding its role in the disease pathogenesis probably will be to label such molecule as “destructive”.

In this scenario, the use of additional distinct and phenotypes can allow additional data analysis and provide some insightful information about how host inflammatory immune response mediators can impact periodontitis outcome. One possible approach is to compare periodontitis variants, such as aggressive and chronic, each one characterized by its unique features, such as early vs late onset and different progression rates.^12^ In such comparison, it is possible to observe variations in RANKL/OPG ratio, which can explain the possible variations between the forms, but the sole comparison of each form with healthy tissue would not allow such inference, despite the “very significant” P value, “more significant” than the aggressive *vs* chronic comparison. Importantly, the comparison on distinct disease forms points to a differential balance in the levels of pro- *vs* anti-osteoclastogenic and pro- *vs* anti-inflammatory mediators as determinants of disease outcome. However, the determination of the “tipping point” that separates homeostasis from pathology may require additional approaches, which will be explored further below.

One may argue that phenotypic variation may not be necessary, since it would be possible to perform some severity disease stratification within an individual disease form, based in clinical readouts such as bleeding on probing, probing depth and attachment loss. However, it is mandatory to consider the intrinsic limitations imposed by periodontitis features, which include the cumulative nature of disease severity, and the uncertainness about disease progression patterns, impose critical limitations to data interpretation.^13^,^14^ Importantly, the definition of active bone resorption is quite complex in periodontitis, which seems to progress via active disease bursts followed by quiescent periods, being the current clinical tools ineffective in the determination of the actual status of disease during the analysis/sampling, and even to determine the progression model.^11^,^14^ Indeed, a 9 mm deep periodontal pocket in a supposed stable/remission state can present distinct features of a 4 mm deep conjectural active site. Indeed, in general, “immediate” parameters such as bleeding on probing and/or probing depth have been found to not be directly correlated with cytokines, irrespectively of its pro-inflammatory (where a positive correlation could be expected) and/or with anti-inflammatory features (where a negative correlation could be expected).^15^–^18^ A similar picture can be observed in periapical lesions, where the lesion size does not necessarily correlates with a “higher” activity signature (i.e. expression of tissue destructive mediators) than smaller lesions (at the moment of sample collection).^19^–^22^

Therefore, the comparison of periodontitis-derived data with other conditions can also support better interpretation of RANKL/OPG ratio association with active or inactive bone resorption. While the definition of active bone resorption is complex in periodontitis, in orthodontic tooth movement such patterns are more straightforwardly distinguishable.^23^–^25^ Categorically, the bone resorption activity is a hallmark of pressure side, and can be comparatively analyzed in the tension side counterpart, where the bone formation activity prevail.^26^–^28^ Such data can provide a theoretical cutoff or threshold value that distinguish presence of absence of active bone resorption, which can be applied to periodontitis or other inflammatory osteolytic conditions, such as periapical lesions, to support additional analysis or assumptions.^22^,^29^,^30^

In an additional example on how the use of distinct phenotypes can provide “biological significance” that can overcome “statistical significance” in providing data interpretation support, let's consider that a family of molecules, collectively called SOCS. SOCS are intracellular proteins that acts as suppressors of inflammatory cytokine signaling, and therefore, are putatively involved in the control of inflammatory response in periodontitis, which ultimately interfere in the RANKL/OPG ratio modulation and in the disease outcome.^21^,^31^ A study demonstrate that SOCS levels are upregulated in diseased tissues when compared with healthy ones, associated with a “very significative” P value (P<0.001).^32^ Similarly to previously mentioned to OPG and anti-inflammatory mediators, one could argue how increased levels of inflammation suppressors, associated to “highly significative” P values (in healthy *vs* diseased tissues comparison), are presented in inflamed and diseased periodontal sites? However, such study also demonstrates that SOCS levels are higher in chronic gingivitis than in chronic periodontitis, which provides some interesting additional biological clue, but such association present a “less significative” P value (P<0.05) than the healthy vs diseased approach.^32^ Based in the initial comparison, it is possible to recognize that SOCS are generally absent in healthy tissues being upregulated in response to inflammation. However, the second scenario allow us to infer that a more pronounced upregulation in chronic gingivitis could suggest a more efficient suppressive feedback, which could account for some phenotypic variation between gingivitis and periodontitis. Even considering that the P value from “healthy *vs* diseased” analysis (P<0.001) is higher than in the “phenotypic variation” comparison (P<0.05), the second comparison may be biologically “more significant” or more relevant for data interpretation.^32^ It is also important to note that oftentimes, a result that indicates statistically significant differences has little or no biological impact. Therefore, it is essential that the researcher knows that although methodologically important in study, above the statistics should be his biological knowledge and interpretation about the results. In this context, it is possible to consider that the “biological significance” can overcome the “statistical significance” in providing support the data interpretation, allowing a broader picture of the immunoregulatory scenario.

Indeed, the analysis of data generated from “phenotypic variation” other than the simple “healthy vs diseased” dichotomy allows a series of correlation analysis that would result in false positive results in the “healthy *vs* diseased” analysis. Please remember that diseased periodontal tissues are characterized by high levels of theoretically destructive elements, such as osteoclastogenic factors (including RANKL) and pro-inflammatory molecules, but also for high levels of supposedly protective elements, such as OPG and anti-inflammatory molecules (such as IL-10), when compared to healthy tissues. Since a high variation in the levels of such molecules is present between health and disease conditions, correlation analysis including samples from both groups presents a high trend to biased “false correlations”.^21^,^32^ It is known from experimental studies (whose importance will be considered in the sequence) that IL-10 induces OPG upregulation and RANKL downregulation. However, a ”healthy *vs* diseased” correlation analysis can result in positive correlations between IL-10 and OPG, but also between IL-10 and RANKL, with “very significative R and P values” (unpublished data). When such correlation is performed observing the “phenotypic variation”, or performed only with a single disease from samples, the positive correlation between IL-10 and OPG is sustained, but with “less significative R and P values”. Additionally, such analysis also reveals that IL-10 and RANKL are non-correlated, being RANKL levels actually correlated with pro-inflammatory mediators (unpublished data); being this data in accordance with IL-10 properties previously mentioned.^21^,^32^ Also, similar patterns (positive correlations between IL-10 and OPG) were observed when lesions are stratified as theoretically active of inactive based in RANKL/OPG threshold, being IL-10 and OPG expression predominant in inactive lesions.^22^,^29^,^30^,^33^

Moreover, when biological events are evaluated such as those mentioned above, it may be naive to trust that a single independent variable would be responsible for the outcome response evaluated. Correlation analyzes may present significant p values between OPG/RANKL with IL-10, but most likely are not the only factors influencing the such ratio values. Therefore, any consideration about the subject that is not based on a broader spectrum of possible influencing factors (independent variables) is naturally weakened, even if based on significant p values. Thus, for biological analyzes involving complex mechanisms, multiple regression models (linear, logistic, Poisson, etc.) could be extremely useful in assessing the impact of a number of independent variables, such as age, gender, ethnicity, oral hygiene, specific clinical variables, presence or absence (and quantity) of a series of inflammatory mediators and pathogenic microorganisms, genetic variants, among others in the analysis of a simple outcome such as having or not having the disease. However, due to the biological complexity involved in this example, the list of independent variables could be infinite if it had no methodological impact either. For regression models to be robust in data analysis, for each independent variable included in the research, in a very simplistic way, 10 to 15 sample units (animals, patients, etc.) should ideally be included in the study. However, in some cases this theory may be beautiful on paper but impossible in practice as it could make a methodologically unviable study due to the large minimum sample size required.

Also, it is mandatory to consider that dichotomization or comparisons based in phenotypic data completely differ from dichotomization or stratification based in random scores frequently attribute to the analysis process. Indeed, it is common to receive in JAOS the submission of papers comprising the use of percentage scores, derived from the quantification of cells positively stained for a given target, such as RANKL. In this virtual scenario (roughly based in submitted papers), score zero refers to 0 to 5% of stained cells, score one refers to 5 to 25%, score two to 26 to 50%, and so on. Therefore, a 25% sample and a 26% sample would receive different scores, while 26% and 50% samples would receive the very same score. It seems that it is not necessary to apply complex statistical tools to realize that some qualitative ‘downgrade’ may not be the best option for the subsequent data analysis, especially when the quantitative data is available. This strategy leads to a weakening of the dependent variable and consequently a less robust and accurate data analysis. When the stratification is necessary, and phenotypic data is not available to guide the stratification, the use of tertiles, quartiles, deciles or cluster analysis can be more adequate than the random assignment of samples into scores or subgroups. Indeed, a cluster analysis demonstrated that the clustering of osteolytic periapical lesions was primarily based in RANKL/OPG,^29^ presenting a high match (>90%) with the inactive/inactive classification based in pressure/tension RANKL/OPG threshold. Additionally, the stratification of the lesions based in RANKL/OPG tertiles reveals a very high match between the high RANKL/OPG tertile and the theoretically active lesions.^30^ At this point, is also mandatory to consider that the use of multiple analysis models can also reinforce the strength of the data, despite the lack of direct influence in P values of independent analysis.

Another non-mathematical tool that can be supporting factor for increasing data strength refers to the combination of different approaches in the study design, such as combining clinical and experimental data. Return to the unclear nature of bone activity or inactivity in periodontitis, and the possibility of determine a theoretical threshold value that distinguish presence of absence of active bone resorption based in tension/pressure sides of orthodontic movement. Despite comprising and valid and interesting approach, one may argue that orthodontic tooth movement features may be not applied to periodontal disease. It is possible to argue that such assumptions are purely associative, and are not definitive demonstration of lesions activity/inactivity status. In this context, experimental models appear as a very important experimental tool do provide non-mathematical/non-statistical “biological significance” support to the clinical data. The possibility of performing longitudinal and controlled analysis, and to implement cause-and-effect experiments, with the induction or inhibition of a given factor (by genetic or pharmacological ways), can provide definitive demonstration of such factor role in a determined condition.

Still in the periodontitis activity/inactivity setting, experimental periodontitis in mice is characterized by sequential stages, where the initial response involves a major influx of inflammatory cells, and rapid and evident bone resorption and a high RANKL/OPG ratio.^11^,^34^–^36^ The subsequent stage is characterized by a minor progression of bone loss, a change in the pattern of immunoregulatory elements and a low RANKL/OPG ratio. The modulation of RANKL/OPG ratio, specifically upregulation of OPG by the selective attraction of immunoregulatory cells, such as Tregs, is associated with the prevention of bone loss.^37^–^40^ Conversely, the inhibition of immunoregulatory feedback and the maintenance of high RANKL/OPG over time result in increased disease severity.^41^ Among the immunoregulatory elements, IL-10, a potent anti-inflammatory molecule which is extensively produced by Tregs, was also demonstrated to inhibit inflammation and to arrest experimental periodontitis progression.^18^,^42^–^44^ Full circle, one of the anti-inflammatory mechanisms of IL-10 comprise the upregulation of SOCS, which in turn limits the action of pro-inflammatory mediators, in parallel with the capacity of IL-10 to upregulate OPG. Taken together, this experimental data suggests that periodontal lesions inactivity may be under the control of IL-10, which in turn upregulate OPG, induces SOCS and consequently limits pro-osteoclastic and pro-inflammatory activity.^32^,^44^,^45^

While experimental data does not provide any kind of additional “statistical significance” to the associative data derived from human studies, including those previously mentioned along this essay, the “biological significance”, despite being numerically unmeasurable, is remarkable. Despite the unprivileged position in the scientific evidence pyramid, the “pre-clinical” research is essential in unraveling mechanistic evidences for biological and pathological processes, and to provide the basis for subsequent clinical interventions.^46^ Mice with opposing maximal and minimal inflammatory responsiveness genotypes and phenotypes present distinct susceptibility/resistance patterns when exposed to periodontopathogens, reinforcing the genotypic impact over the phenotype unraveled under “exposure” conditions,^47^ PMID.^48^ The marked susceptibility of IL-10 deficient mice to periodontitis development^43^ supported the investigation of genetic variants that modulate IL-10 levels in humans,^18^ and its role as risk factors for periodontal disease development with potential application for diagnosis and treatment planning. The possibility of modulating the inflammatory environment nature in periodontium by IL-10, derived from Tregs recruitment,^37^–^40^ was demonstrated to the useful in pre-clinical models, which support its potential application in future clinical trials. The discovery of RANKL/OPG axis in experimental models leaded to the development of OPG-mimicking/analogues therapeutics currently used in humans for diverse bone pathologies.

Finally, we reiterate that this piece (similarly to the Amrhein's Nature assay) are not advocating a ban on P values or statistical measures, which remains essential. However, it is crucial to consider that statisticians are not mystical magical creatures and therefore there are no statistical miracles to marvelously overcome poor experimental design.

Thus, despite any major change in the way of P values or statistical tests are applied and interpreted, we must consider that the attention to the proper study design may have an immediate and positive influence in the numerically unmeasurable data “biological significance”.
